# Focusing attention on others’ negative emotions reduces the effect of social relationships on children’s distributive behaviors

**DOI:** 10.1371/journal.pone.0295642

**Published:** 2024-02-07

**Authors:** Minjung Cha, Hyun-joo Song

**Affiliations:** Department of Psychology, Yonsei University, Seodaemun-gu, Seoul, Republic of Korea; Central University of Finance and Economics, CHINA

## Abstract

The present study investigates whether directing five- to six-year-old children’s attention to hypothetical resource recipients that included familiar and non-familiar people would affect their favoritism toward a familiar person, as reflected in how they allocated resources. In Experiment 1, we instructed participants to give one of several stickers to another person or keep all the stickers for themselves. Under the control conditions, participants more frequently gave stickers to friends than to non-friends. However, when asked about others’ emotions, they distributed stickers equally among friends and non-friends. Therefore, focusing on others’ thoughts reduced participants’ favoritism toward friends. Experiment 2 tested whether focusing on both emotional valences would affect favoritism toward a familiar person, as reflected in children’s resource distribution choices. Experiment 2 was identical to Experiment 1, except we asked participants about the other person’s emotional valence. When asked about others’ negative emotions, participants distributed the stickers equally between themselves and others. However, when asked about others’ positive emotions, they distributed more stickers to friends than to non-friends. Neither others’ emotional valence nor group status affected the perceived intensity of their emotion or the participant’s emotional state. These results suggest that children’s favoritism toward friends can be reduced by encouraging them to think about others’ negative emotional states.

## Introduction

Adults prefer those with whom they share close relationships, such as friends, over those with whom they do not [1–4]. Even when adults are randomly divided into two groups without meeting other group members, they prefer to identify with the in-group, accentuate its positive qualities, and allocate more resources to it [2]. Likewise, a recent study based on the dictator game indicates that such favoritism is more likely to arise in situations of low resources or constraints [5].

This tendency is observable even in childhood [6–9]. As children age, they become more conscious of social connections and the potential consequences of deviating from social interactions, such as social exclusion [10, 11]. By adulthood, favoritism toward familiar individuals is deeply ingrained, leading to the belief that interventions to mitigate such bias should start in childhood [12]. Discovering strategies to lessen these tendencies in childhood can offer insights into why children exhibit these tendencies and may reveal factors that contribute to the development of fairness in childhood.

Researchers have investigated how social relationships affect resource distribution among children using a resource allocation paradigm in which children make decisions to distribute resources among themselves and others. In the dictator game paradigm, one assumes the role of the proposer, who determines how to allocate the resource, while the other assumes the role of the responder, who receives the resource. The players must choose between a fair distribution of resources with equal payoffs and an unfair alternative distribution that may be profitable or detrimental to them. In such a situation, children typically offer more material support to those with whom they have a close relationship than to those with whom they do not have close relationships. Eight-year-old children were more likely to distribute resources to classmates than to children in other classes [13]. This effect holds even for relatively younger age groups. Another study found that three- to five-year-old white children allocated more resources to other white children of the same gender than to children of different genders and races [14]. Recent findings indicate that preschoolers make decisions on how to distribute their resources based on their social relationships, such as friendship [15–17]. A recent study on children’s resource allocation revealed that by age 5, children begin to favor their friends over non-friends when giving resources [16]. Interestingly, another study indicated that preschoolers’ preference for sharing with peers is not influenced by strategic considerations like the expectation of reciprocity [17].

The current research primarily concerns whether the favoritism toward close relationships exhibited in children’s sharing behaviors can be lowered in some circumstances. Because such favoritism toward familiar others is frequently associated with negative social consequences such as increased hostility toward individuals of different races [18], and a diminished likelihood of exhibiting moral behavior [19], an extensive body of research has concentrated on improving attitudes toward individuals whom one lacks a close connection [3, 20–22]. Several findings suggest that negative attitudes toward unfamiliar individuals might arise from perceiving them as less human, leading to the attribution of fewer mental states to those who are less familiar [23]. In a similar vein, preschool children struggle to reference the mental states of out-groups [24, 25]. Considering such evidence, mentalization, involving the focus on the cognitive and affective aspects of another individual [26], has been suggested as a method for promoting prosocial behavior toward unfamiliar peers in children [25].

The specific aspects of mentalization that may produce this effect however, remain unclear. Researchers widely believe that comprehending others’ emotions significantly influences empathic and prosocial behaviors, often more than understanding their thoughts [27, 28]. In line with this assumption, affective perspective-taking (identifying others’ feelings) is more likely to promote empathic arousal and helping behaviors in adults than cognitive perspective-taking [29]. Some evidence on children suggests a link between children’s altruistic actions and their ability to identify others’ feelings [30] but no prior studies have examined the relative role of affective and cognitive perspective-taking in enhancing children’s prosocial actions within a single study. Therefore, our current research specifically examines a subset of cases in which concentrating on other’s emotional or cognitive mental states produced differing outcomes in reducing favoritism among children.

### Background literature

Previous research has examined various ways to reduce children’s favoritism toward close relationships; one way is through direct intergroup contact [26, 31–33]. For example, children in racially diverse communities exhibit less racial prejudice than those in racially homogeneous communities [34, 35]. Similarly, researchers have found a positive correlation between the number of non-white friends of eight- to 11-year-old white children and these children’s attitudes toward non-white groups [36] and an inverse relationship and negative correlation between children’s preferences for friendships with children of the same race and intergroup contact [37].

While direct contact is a strong option for reducing favoritism toward those with whom one has close ties, indirect contact is also effective. One way to achieve indirect contact is through mentalization, which refers to focusing on another person’s mental states, such as their emotions, thoughts, and desires [38, 39]. Mentalization enables children to consider other people’s perspectives and understand their emotions, thoughts, and intentions. Asking children to consider others’ psychological states significantly increases their prosocial behavior toward others. For example, in a previous study, researchers found that asking eight- to 13-year-old children to think about an out-group member’s feelings created stronger intentions to help that member [40]. Another study found that asking five- to six-year-old children to consider the thoughts and feelings of immigrants resulted in them more evenly distributing resources between themselves and an immigrant child [25]. Even Jewish and Palestinian children, who come from cultures with mutual animosity, expressed increased empathy toward each other’s ethnic groups through classroom interventions that include empathy and perspective-taking training sessions [41].

However, there has been relatively little research on the mechanisms by which such mentalization interventions operate. One previous study studied multiple elements (e.g., perspective-taking and empathy) of intervention programs designed to modify intergroup attitudes, but it did not determine which factors were the most salient [41]. Similarly, another study demonstrated that considering an out-group member’s psychological state significantly reduces in-group bias; however, that study did not differentiate types of mentalization according to their effectiveness [25]. Would mentalization directed at others’ thoughts versus their emotions, have a different or similar relationship with children’s prosocial actions toward others?

Mentalizing regarding others’ emotions and mentalizing regarding others’ thoughts are related constructs with a modest degree of intercorrelation [42]. However, emotion mentalization may more robustly foster prosocial actions than cognitive mentalization, because the activation of emotional mentalization primarily triggers affective representations of others, which closely aligns with the concept of "empathizing" [43]. Empathy can lead to a greater willingness to engage in positive intergroup interactions and to see members of other groups as individuals with their own unique experiences and perspectives [44, 45]. This is particularly true for tasks such as forming social partnerships and minimizing relationship conflict [46, 47]. In a similar vein, a meta-analysis has found that empathy is a significantly more effective mediator than knowledge in reducing bias between groups [48].

This tendency that emotional mentalization better fosters prosociality also holds among children, and previous evidence from studies on children aligns with that from the above-described studies on adults. Mentalizing others’ emotions improves children’s attitudes toward people of other races [49] and ameliorates their anti-immigrant attitudes [50]. Higher empathy is also associated with earlier prosocial behavior; children with greater empathy are more willing to share resources, help those in need, and comfort those in distress [51, 52]. In contrast, low empathy in childhood is associated with bad peer relationships, violence, and bullying [53]. However, empirical evidence for the effect of mentalizing others’ thoughts on children’s attitude toward others is less consistent. Children’s ability to understand others’ emotions predicts their tendency to assist victims and prevent bullies from threatening others, whereas their ability to comprehend others’ cognitive perspective does not [54].

Furthermore, previous studies have suggested that the effectiveness of mentalizing others’ emotions in improving attitudes toward those people may depend on emotional valence. Studies have consistently demonstrated that relating to others’ negative emotions (e.g., sadness) improves attitudes toward others [42, 55, 56], but it is debatable whether relating to other’s positive emotions has the same effect. One line of research has found that mentalizing others’ positive emotions promotes prosocial behavior in children of various age groups [57, 58], while another has suggested that there is no effect. For example, a correlation was found between nine-year-old children’s emotional reactions to others’ negative emotions and less disruptive behavior [59]. However, there was no such relationship for emotional responses to others’ positive feelings. Similarly, a strong correlation was discovered between responding to negative emotions and social capacity in elementary school children but no such correlation was found for positive emotions [60].

### The current study

Despite the abundance of evidence that bias-reduction interventions can be successful, further research is needed to understand why certain interventions are more effective than others. This study aims to fill this gap by 1) separately examining the effects of considering others’ thoughts and emotions on favoritism toward a close friend and 2) examining whether the valence of the emotions that people mentalize affects this favoritism. We employed the approach of familiarity classification based on social relationships [61] to ensure that the children could readily distinguish between familiar and non-familiar others.

We conducted this research in two parts. Experiment 1 tested the hypothesis that calling children’s attention to another child’s emotional state reduces their favoritism toward familiar children more than calling attention to the child’s cognitive state. Specifically, we asked children how many resources they would give to their best friend or a stranger child after telling them a story about the other child’s emotions or thoughts. Experiment 2 tested the hypothesis that calling attention to another child’s negative emotional state reduces favoritism toward a close friend more than calling attention to the child’s positive emotional state. We explored these questions with five- to six-year-old children. Previous research has demonstrated that children in this age range have strong favoritism toward friends [7, 13], which includes a proclivity to increase prosocial behavior toward strangers by mentalizing their psychological states [25].

## Experiment 1

### Method

#### Participants

The participants were 106 South Korean children (52 females, 54 males; mean age = 71.9 months; age range = 60.1–82.3 months). We recruited the children by posting advertisements in online parenting communities and distributing leaflets describing infant development research at a public health center. Each child’s parent or guardian gave written informed consent. This study was conducted in accordance with the ethical guidelines and approval of the Institutional Ethics Review Board at Yonsei University, South Korea. We excluded four additional children from the analysis because they refused to answer the experimenter’s questions (*n* = 3) or were inattentive (*n* = 1). The sample size for this study was determined to achieve 80% power based on prior studies [13, 62] in order to detect a condition effect with three levels on the difference in children’s egalitarian choice towards in-group versus out-group individual. We performed an a priori power analysis [G*Power 3.1.9.4; 63] for a binary logistic regression model with an alpha of .05, and obtained that at least 92 participants to detect a significant effect of condition.

#### Procedure and measures

Before the experiment, the participants were asked to select a best friend of the same gender as themselves and provide their first names. Then, the experimenters presented the participant with the name of a stranger and stated that their gender was the same. Then, the child participants completed two trials (“friend” and “stranger”), each of which consisted of story and distribution phases. During the story phase, children heard stories about their best friend or a child stranger. Then, during the distribution phase, we asked them to choose whether to share a sticker with their best friend or the stranger. We randomized the trials’ order across participants. Although we told the same stories about their best friend and an unfamiliar child across the two trials, none of the participants found it strange to hear the same story twice. All procedures were carried out in Korean.

*Story phase*. We first asked children to name their best friend of the same gender, who served as an in-group member, and then we provided the name of a child stranger of the same gender to serve as an out-group member. The experimenter then told the participant a story in which the best friend or the stranger went to a store to buy stickers but found that there were no stickers left in the store. In the control condition, children learned that the protagonist looked for another place to buy stickers, and the experimenter asked where the protagonist might have gone, as in the following story:

“[Best friend’s name or the stranger’s name] went to a store a few days ago because they wanted to buy some stickers. However, there weren’t any at that store, so they looked for another place to buy stickers and made a face like this (experimenter shows a picture of a neutral face to participant). Let’s think about where they searched for stickers (experimenter pauses for four seconds). Where do you think they went?”

In the emotional condition, the children learned that the protagonist felt sad. Then, the experimenter asked them to infer how sad the protagonist might have felt, as in the following story:

“[Best friend’s name or the stranger’s name] went to a store to buy some stickers a few days ago. However, there were no stickers left. They felt very sad, so they made a sad face like this (the experimenter shows a picture of a sad face to the participant). Let’s think about how sad they felt for a second (experimenter pauses for four seconds). How sad did they feel?”

In the cognitive condition, the children learned that the protagonist had thought about obtaining stickers. Then, the experimenter asked them to infer how much they might have thought about it, as in the following story:

“[Best friend’s name or the stranger’s name] went to a store to buy some stickers a few days ago. However, there were no stickers left, so they thought about how to get the stickers. They made a thinking face like this (experimenter shows a picture of a neutral face to the participant). Let’s think about how much they thought about it for a second (experimenter pauses for four seconds). How hard do you think they thought?”

The pictures accompanied by the stories were identical in all three conditions, with the exception of the protagonist’s facial expression. The emotional condition picture depicted the protagonist with a crying expression, whereas the control and cognitive condition pictures depicted the protagonist with a neutral facial expression.

The experimenter intended for the questions at the end of each condition’s priming stories to be answered with an open response, and children had four seconds to generate an answer. If they did not respond the first time, the experimenter repeated the question before advancing to the next step in the procedure.

*Distribution phase*. After the story phase, children were asked to participate in a mini-dictator game. The mini-dictator game gives players two predetermined choices for distributing the resources, as opposed to the usual dictator game, which requires players to allocate any number of the total resources. Such a method has the benefit of being simple enough for children to perform [13]. In our study, children were asked to choose between egalitarian and selfish offers. We presented participants with two stickers and asked them to allocate the stickers between themselves and another person. There were two trials, and we asked children to give stickers to their best friend in the first trial and to the stranger in the second trial. The order in which the best friend and the stranger were introduced was counterbalanced. Children could choose from only two predicted options: 1) take both stickers for themselves (selfish offer) or 2) take one sticker for themselves and give the other sticker to the other child (egalitarian offer). The children were tested with an experimenter in a room separate from their parents. The experimenter informed the children about the study and explained to them that the stickers they selected would be given to the other individual later. We coded the first response as unequal distribution and the second as equal distribution. We then compiled and analyzed the frequencies of children who selected the equal distribution option in both trials.

*Manipulation check*. To ensure that children reflected on their emotional and cognitive states in the Emotional and Cognitive conditions, respectively, we classified the total number of mental state words they used in the Emotional and Cognitive condition. We classified the mental state content of children’s descriptions using a coding scheme similar to McLoughlin and Over’s (2017) classification system. Words were categorized as referring emotional states if they related to a character’s emotions (such as "to be sad," "to be disturbed," or "to be annoyed"), and cognitive states if they referred to a character’s thoughts ("to think," "to consider"). As a result, all participants (38 of 38 children) in the Emotional condition reported emotional state-related responses, while all participants (32 of 32 children) in the Cognitive condition reported cognitive state-related responses. These results indicate that children reflected on the emotional and cognitive states of the individual they were asked about.

### Results

**Fig 1:**
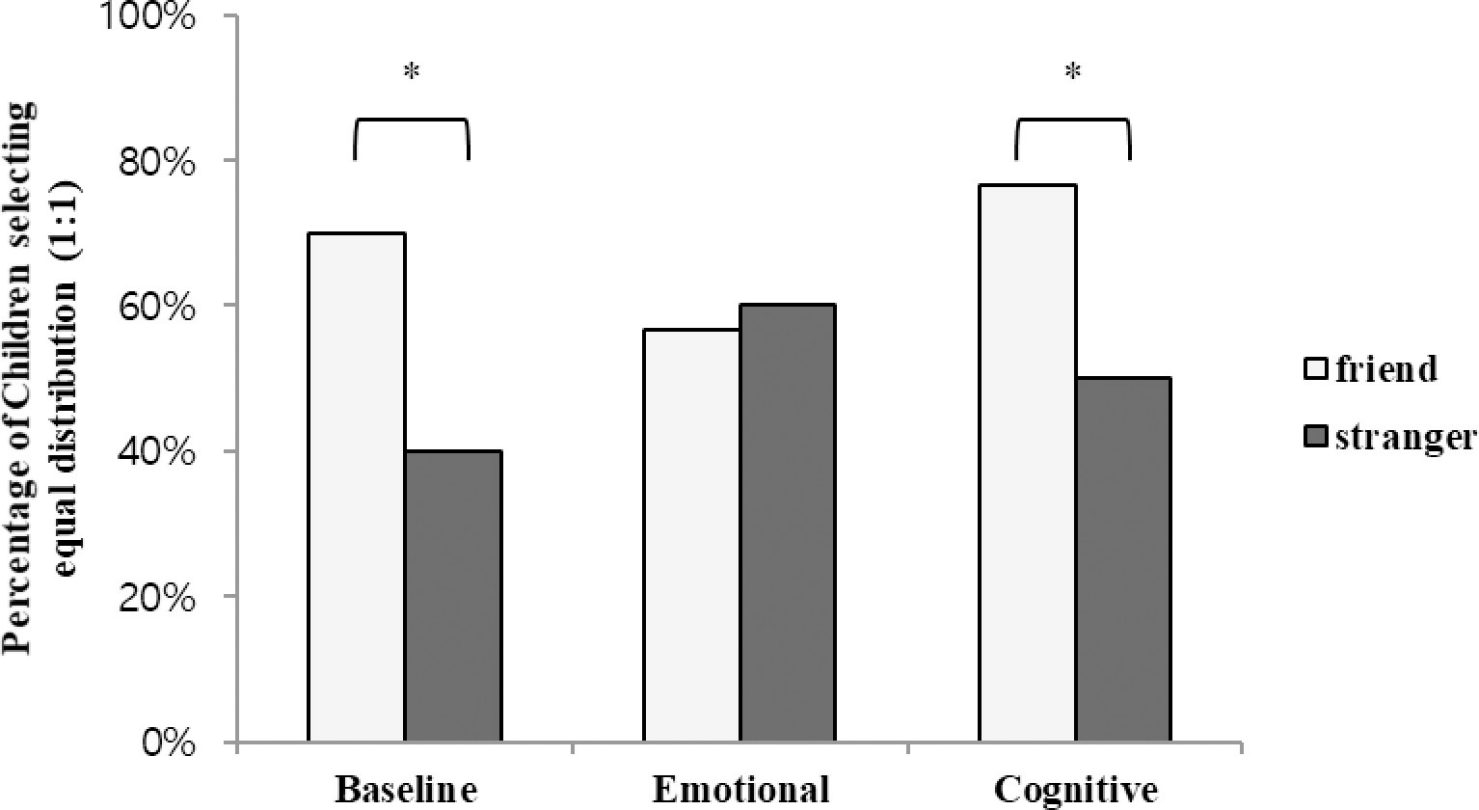
Children’s choice of resource distribution to familiar and unfamiliar others (Experiment 1).

Preliminary analyses revealed no effects of age or gender. Therefore, we utilized the complete dataset for all subsequent analyses. The purpose of the analysis was to determine the effect of the mentalization condition (between-subjects) and the familiarity of the recipient (within-subjects) on the children’s selection of equal distribution. A binary logistic regression with condition (control, emotion, and cognitive) and familiarity of the recipient (friend and stranger) as predictors showed that the recipient’s familiarity significantly predicted children’s selection of equal distribution (Wald *X*^*2*^ (1, *N* = 212) = 14.07, *p* < .001). However, the conditions in the study did not significantly predict children’s selection of equal distribution (Wald *X*^*2*^ (2, *N* = 212) = 1.59, *p* = .45). There was a significant interaction effect between familiarity and condition (Wald *X*^2^ (2, *N* = 212) = 9.94, *p* = .01) (Fig 1).

More specifically, a 2 (conditions: control and emotion) × 2 (familiarity of recipient: friend and stranger) binary logistic regression found a significant interaction effect (Wald *X*^2^ (1, *N* = 148) = 9.47, *p* < .001). However, there was no significant interaction effect for an alternative 2 (conditions: control and cognitive) × 2 (familiarity of recipient: friend and stranger) binary logistic regression analysis (Wald *X*^2^ (1, *N* = 136) = .87, *p* = .35).

We conducted a planned comparison test of children’s resource distribution choices in each condition. In the control condition, 75% of children (27 of 36) shared equally with their friend and 33% (12 of 36) shared equally with a stranger, which indicates a preference for friends in resource sharing. A binary logistic regression with familiarity as a within-subject factor revealed a significant difference in children’s equal resource distribution between a friend and a stranger (Wald *X*^2^ (1, *N* = 72) = 16.13, *p* < .001). However, in the emotional condition, we found a reduced preference for friends, with 58% of children (22 of 38) choosing to share equally with a friend and choosing to share equally with a stranger. Moreover, there was no significant difference in children’s equal resource distribution between a friend and a stranger in this condition (Wald *X*^*2*^ (1, *N* = 76) = .00, *p* = 1.00). In the cognitive condition, a preference for friends continued to manifest, with 78% of children (25 of 32) choosing to share equally with a friend and 53% of children (17 of 32) choosing to share equally with a stranger. This difference was also statistically significant (Wald *X*^*2*^ (1, *N* = 64) = 4.78, *p* = .03).

We conducted additional analyses to compare children’s equal resource distribution to friends across the three conditions. The results indicate no significant difference in the distribution of stickers to the friends across the conditions (Wald *X*^*2*^ (2, *N* = 106) = 3.97, *p* = .14). Further analyses confirmed that neither the emotion nor cognitive condition significantly differed from the control condition in the proportion of children who selected equal distribution to friends (emotion vs. control: Wald *X*^2^s < 2.40, *ps >* .12).

Likewise, we performed an analysis to compare children’s equal resource distribution to strangers across conditions. The results indicate no significant difference in the distribution of stickers to strangers across the conditions (Wald *X*^*2*^ (2, *N* = 106) = 4.81, *p* = .09). However, further analyses revealed that the emotional condition significantly differed from the control condition in the distribution of stickers to strangers (Wald *X*^2^ (1, *N* = 74) = 4.39, *p* = .04); however, the cognitive condition did not (Wald *X*^2^ (1, *N* = 68) = 2.67, *p* = .10).

### Discussion

Experiment 1 demonstrates that mentalizing others’ emotional states significantly increased participants’ resource allocation to strangers. For example, children chose to share stickers with friends more frequently than with strangers in the control condition; however, this tendency ceased when participants considered their partner’s emotional state but not their thoughts. These results replicate previous findings that children display a preference for friends in resource sharing and add to the evidence that such attitudes are affected by mentalization. Mentalizing others’ emotions, but not thoughts, effectively reduces preference for friends. This finding is consistent with previous evidence that children act more prosocially when they have more explicit information about others’ internal states [64].

## Experiment 2

Experiment 2 examined whether the valence of the emotion about which children mentalized would affect their favoritism toward friends. The existing literature regarding how focusing on negative emotions affects children’s prosocial behavior is largely consistent [55, 65, 66]. For example, children who are attentive to others’ distress exhibit increased prosocial behavior [67]. In contrast, there is an inconsistent association between calling attention to positive emotions and prosociality [59, 60, 68]. In accordance with previous work, we expected that calling attention to negative emotions would weaken children’s bias toward friends in resource distribution contexts. Therefore, Experiment 2 included the following three conditions—positive emotion, negative emotion, and control—to test this hypothesis.

Furthermore, Experiment 2 made several modifications to the procedure to address some questions that arose from Experiment 1. First, there is an alternative interpretation for why the emotional condition produced different results than the other two conditions in Experiment 1; individuals detect emotions more readily than non-emotions when they process social information [69]. Therefore, it might have been more cognitively demanding for participants to reason about their partner’s goal location in the control condition and their cognitive effort in the cognitive condition than their emotional state in the emotional condition. The processing demands imposed by non-emotion information might have hindered the reduction of bias toward friends in the control and cognitive conditions. The children’s responses to the open-ended questions could provide insight into this interpretation.

Children produced more diverse responses in the control and cognitive conditions than in the emotional condition. Simple answers such as “a lot” or “a little” were less frequent in the control (61%) than the emotional condition (70%). In the cognitive condition, children often came up with detailed answers such as “12 hours” or “five times” (33%), but in the emotional condition, none generated numerical responses. For the control condition, most children (94%) mentioned various locations (79% mentioned stores such as a supermarket, convenience store, and stationery shop, and 15% mentioned other places such as a friend’s house, theater, and forest). These data suggest that children might have mentally searched through more diverse response options in the control and cognitive conditions than in the emotional condition; selecting from diverse choices might have been cognitively demanding. To address this issue, the control condition of Experiment 2 did not include a story about the partner; as such, the cognitive demand of processing non-emotion information would theoretically not be an issue in this condition.

Second, children’s emotions while observing their partner’s positive or negative emotions could affect their sharing intentions. For example, simply observing another person’s emotions activates the brain regions associated with empathy [70]. In addition, numerous studies indicate that empathy plays a significant role in prosocial behavior [44]. Therefore, our participants’ emotional experiences in the emotional conditions could have affected their sharing propensity. To address this possibility, in Experiment 2, we assessed participants’ emotions in the two emotional conditions.

Third, children’s sharing intentions could be affected by the difference in perceived emotional intensity between friends and strangers. Adults interpret in-group members’ emotions more accurately than those of out-group members [71]. Similarly, children may judge familiar and unfamiliar members’ emotional experiences differently. For example, if participants infer that strangers’ happiness or sadness is less intense than that of friends, they may be less likely to distribute stickers equally to strangers. As such, in Experiment 2, we assessed the intensity of the emotions that the participants thought their partners were experiencing in the positive and negative emotion conditions.

Fourth, in Experiment 1, we asked participants to choose whether to share stickers with a partner who had failed to obtain stickers in the previously heard stories. Thus, it is unclear whether the effect of emotion observed in Experiment 1 would be specific to the recipient’s situation of having scarce resources. To address this issue, we examined whether children’s focus on others’ emotional experiences in social situations not involving stickers would also reduce bias toward friends in a subsequent sticker-sharing task.

### Method

#### Participants

The participants were 95 children (49 females, 46 males; mean age = 71.4 months; age range = 60.6–83.8 months). We recruited the children by posting advertisements in online parenting communities and distributing leaflets describing infant development research at a public health center. Each child’s parent or guardian gave written informed consent. This study was conducted in accordance with the ethical guidelines and approval of the Institutional Ethics Review Board at Yonsei University, South Korea. We excluded two participants from the analysis because they refused to respond to the experimenter’s questions (*n* = 1) or were inattentive during the experiment (*n* = 1).

#### Procedure and measures

The procedures and measures used in Experiment 2 were similar to those used in Experiment 1, but there were several differences. First, in the control condition, we did not tell participants a story about their partner; they simply completed the same resource allocation task as in Experiment 1. Thus, they did not need to consider their partner’s psychological states. Second, there were two types of emotional conditions—positive and negative—in which we asked participants about their partner’s positive or negative emotions, respectively. Third, unlike Experiment 1, the stories were not about the partners’ lack of stickers. Fourth, we measured the perceived intensity of the partners’ and participants’ emotions in the emotional conditions at the end of the experiment.

*Story phase*. We told the participants stories about their best friend or a stranger child, except for those in the control condition, for which there was only a distribution phase. In the positive emotion condition, we presented the following story to participants:

“[Best friend’s name or the stranger’s name] went to the playground the other day and had so much fun with their friends. They felt so good and made a happy face like this (experimenter shows a picture of a happy face to the participant). Let’s think about how happy they were (experimenter pauses for four seconds). How happy do you think they were at this time?”

In the negative emotion condition, the story was as follows:

“[Best friend’s name or the stranger’s name] went to the playground the other day and did not have much fun with their friends. They felt so sad and made a sad face like this (experimenter shows a picture of a sad face to the participant). Let’s think about how sad they were (experimenter pauses for four seconds). How sad do you think they were at this time?”

*Distribution phase*. The participants completed the resource allocation trials with the hypothetical best friend or stranger. After completing this task, participants in the positive and negative emotion conditions were asked to infer the intensity of their partner’s emotions.

*Measurement of perceived emotional intensity*. We assessed perceived emotional intensity using stories as adapted from Mcloughlin and Over (2019). In the positive emotion condition, we showed participants a drawing of a penguin or a dinosaur (in random order). We then told the following story, which included positive occurrences that resulted in the protagonist experiencing happiness:

“[Best friend’s name or the stranger’s name] drew this picture a few days ago and was complimented by their teacher for their excellent drawing. How do you think they felt at that time?”

Participants responded to the final question using a four‐point scale, ranging from 1 (“*neither happy nor sad*”) to 4 (“*very happy*”).

In the negative emotion condition, we showed participants a drawing of a penguin or a dinosaur (in random order) that was torn in the middle. Then, we told them the following story, in which the protagonist felt sad as a result of an unpleasant event that occurred:

“[Best friend’s name or the stranger’s name] drew this picture a few days ago, and someone tore the drawing when they were not looking. How do you think they felt at that time?”

Similarly, participants reported how they perceived the protagonist’s sadness on a four‐point scale ranging from 1 (“*neither happy nor sad*”) to 4 (“*very sad*”).

*Measurement of participant’s emotions*. Finally, we measured participants’ emotional experiences at the end of the procedure for the two emotional conditions. We assessed whether their emotions changed after hearing emotion-relevant stories and influenced their sharing intentions. The experimenter showed participants pictures of a happy face, a neutral face, and a sad face and asked them to choose a face consistent with their emotions, as in the following:

“Here are three faces showing different feelings: (The experimenter points to a picture of a happy face) this is a happy face, (The experimenter points to a picture of a neutral face) this face means that you are neither happy nor sad, (The experimenter points to a picture of a sad face) and this is a sad face. How do you feel right now? Can you point to the picture that describes your feelings?”

We coded response selections as follows: sad face = 1, neutral face = 2, and happy face = 3.

*Manipulation check*. To ensure that the children reflected on their emotional and cognitive states in the Positive and Negative emotional conditions, we classified the total number of positive and negative emotional state words they used in the two conditions. We classified the mental state content of children’s descriptions using a coding scheme similar to McLoughlin and Over’s (2019) classification system. Words were categorized as referring to positive emotional states if they related to an individual’s positive emotions (such as “to be happy,” “to have fun”), and negative emotional states if they referred to an individual’s negative emotions (“to be angry,” “to be sad”). As a result, all participants (31 of 31 children) in the Positive Emotional condition reported positive emotional state-related responses. Likewise, all participants (33 of 33 children) in the Negative Emotional condition reported negative emotional state-related responses. These results indicate that the children reflected on the positive and negative emotional states of the individual.

### Results

**Fig 2:**
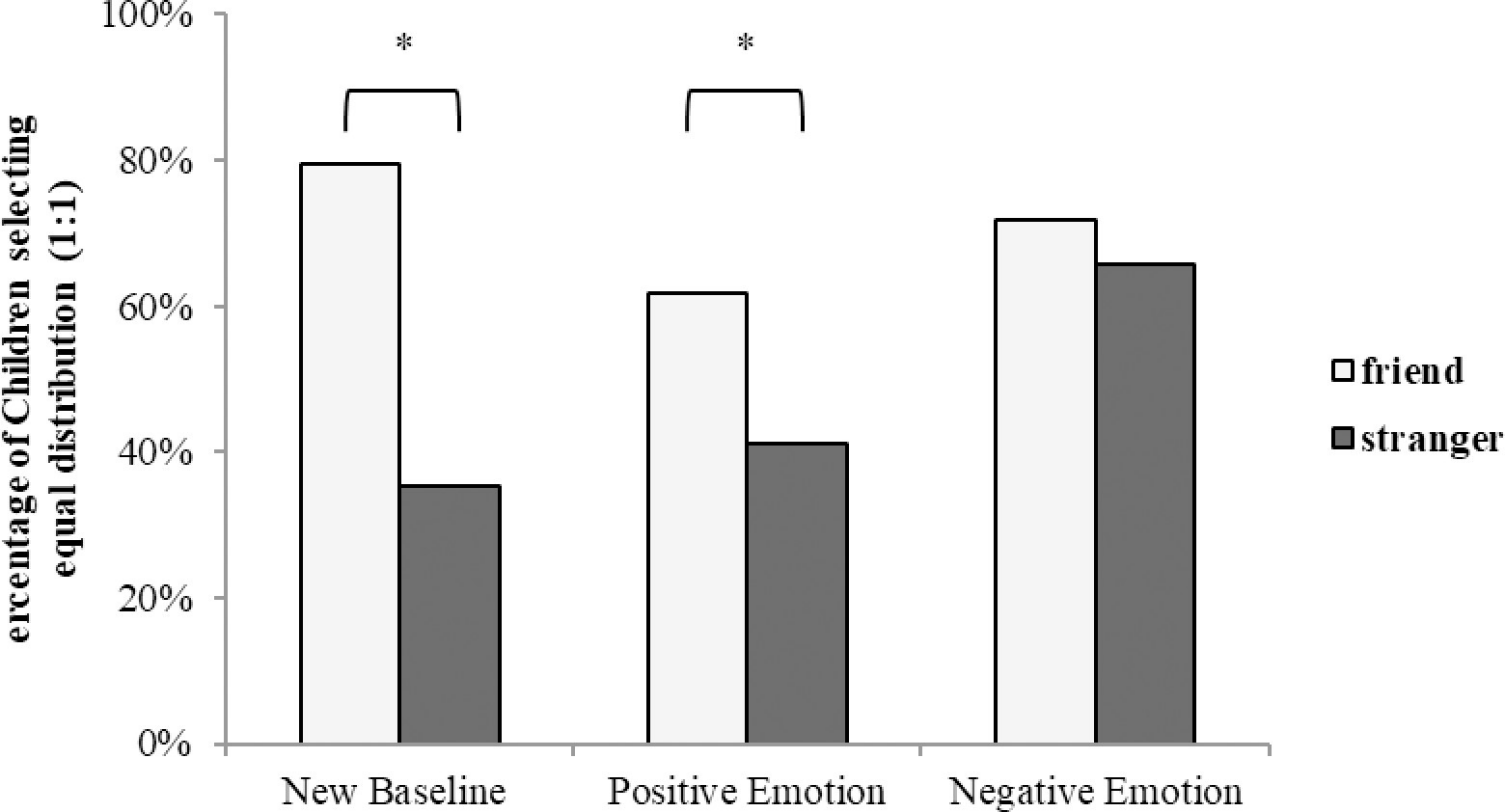
Children’s choice of resource distribution to familiar and unfamiliar others (Experiment 2).

As with Experiment 1, we combined the analyses across gender and age, as no significant differences occurred between these groups. We used binary logistic regression to analyze the selection frequency of the equal distribution option. The predictors included the condition (control, positive emotion, and negative emotion; between-subjects) and familiarity of the recipient (friend and stranger; within-subjects). The results indicate that familiarity with the recipient significantly predicted children’s selection of equal distribution (Wald *X*^*2*^ (1, *N* = 190) = 12.07, *p* < .001). However, the condition did not significantly predict children’s selection of equal distribution (Wald *X*^*2*^ (1, *N* = 190) = 1.69, *p* = .43). Nonetheless, there was a significant interaction effect between familiarity and condition (Wald *X*^2^ (2, *N* = 190) = 7.06, *p* = .03) (Fig 2).

More specifically, a 2 (conditions: control and positive emotion) × 2 (familiarity of recipient: friend and stranger) binary logistic regression found no significant interaction effect (Wald *X*^2^ (1, *N* = 124) = .21, *p* = .64). However, there was a significant interaction effect in the alternative 2 (conditions: control and negative emotion) × 2 (familiarity of recipient: friend and stranger) binary logistic regression analysis (Wald *X*^2^ (1, *N* = 128) = 5.70, *p* = .02).

We then conducted a planned comparison test of children’s equal resource distribution across conditions. In the control condition, children showed a bias toward friends: 74% of children (23 of 31) chose to share equally with friends, whereas 39% (12 of 31) decided to share equally with strangers (Wald *X*^2^ (1, *N* = 62) = 9.04, *p* < .001). Similarly, in the positive emotional condition, children also displayed bias toward friends: 61% of children (24 of 31) chose to share equally with friends, whereas 35% of children (16 of 31) decided to share equally with strangers (Wald *X*^*2*^ (1, *N* = 62) = 6.92, *p* = .01). However, in the negative emotional condition, the bias toward friends ceased—67% of children (22 of 33) chose to share equally with friends and strangers (Wald *X*^*2*^ (1, *N* = 66) = .00, *p* = 1.00).

Additional analyses compared children’s equal resource distribution with friends across the three conditions. There were no significant differences in the distribution of stickers in this case (Wald *X*^*2*^ (2, *N* = 95) = .49, *p* = .78); the outcomes of neither the positive nor negative emotional condition differed from that of the control condition (Wald *X*^2^s < 1). We also compared children’s equal resource distribution among strangers across the three conditions. The condition had a significant effect on the distribution of stickers to strangers (Wald *X*^*2*^ (2, *N* = 95) = 6.50, *p* = .04). The negative emotional condition differed significantly from the control condition in the frequency of equal distribution to strangers (Wald *X*^*2*^ (1, *N* = 95) = 4.88, *p* = .03), whereas the positive emotional condition did not (Wald *X*^2^ (1, *N* = 62) = 0.00, *p* = 1.00).

Table 1 shows the perceived intensity of the hypothetical best friends’ or strangers’ emotions and participants’ perceptions of their own emotional experiences. There was no significant difference in the perceived intensity of happiness or sadness between friends and strangers in the positive or negative emotional condition (*t*s < 1.20, *p*s *>* .25). Further, participants’ perceptions of their own emotions did not differ significantly between the positive and negative emotional conditions (*t*(62) = .37, *p* = .71).

**Table 1:** Means and standard deviations of perceived emotion variables (Experiment 2).

Variable	*M*	*SD*
Perceived happiness of familiar others (positive emotional condition)	3.52	0.93
Perceived happiness of unfamiliar others (positive emotional condition)	3.23	0.88
Perception of own emotions (positive emotional condition)	2.77	0.43
Perceived sadness of familiar others (negative emotional condition)	3.48	0.76
Perceived sadness of unfamiliar others (negative emotional condition)	3.45	0.87
Perception of own emotions (negative emotional condition)	2.73	0.57

Finally, we examined the children’s responses to the open-ended questions posed at the end of the priming stories in the emotional conditions. We aimed to discover whether differences in processing the negative and positive priming might have contributed to the findings. The children’s most frequent responses were simple one- or two-word answers regarding the levels of emotions (e.g., “a lot,” “a little”) in both the positive (92%) and negative (91%) conditions. Thus, we found no indication that the cognitive demand for processing positive and negative emotional stories might have differed.

### Discussion

In the control condition without the story phase, children tended to favor friends over strangers in distributing resources. This result allowed us to exclude the possibility that Experiment 1’s results were due to differences in cognitive demands for processing non-emotion versus emotion information during the story phase. The mentalization of others’ negative emotional states significantly increased participants’ resource allocation to strangers. Interestingly, when children’s attention was directed to others’ positive emotional states, they did not choose to share equal amounts of resources with strangers. These results suggest that children’s attitudes toward unfamiliar others rely on their partners’ emotional valence.

Notably, the influence of attending to negative emotions (even in situations unrelated to a lack of stickers) reduced bias toward friends in the sticker distribution task. These findings suggest that directing children’s attention to others’ negative emotions to reduce bias toward familiar others can readily be applied in diverse situations. Furthermore, this effect was not a product of the difference in how children perceived the intensity of friends and strangers’ sadness or how children perceived their own emotions.

## General discussion

The children in the current research exhibited less bias against strangers when encouraged to think about the strangers’ negative emotional states. In Experiment 1, children tended to share resources evenly with strangers when directed to focus on their partners’ sadness but not when focusing on their partners’ thoughts. Experiment 2 replicated the results of Experiment 1 and revealed that attending to strangers’ sadness, but not happiness, can reduce a preference for friends in resource sharing. These findings strongly support the argument that bias based on social relationships may indeed change as a function of the psychological states of others to which children pay attention. The current research is the first attempt to examine how certain types of mentalization about others’ psychological states affect bias toward friends and strangers in children. Considering another person’s negative emotional state appears to be a key mechanism underlying the reduction in children’s favoritism toward those with whom they have close relationships.

It is unclear whether the current findings also pertain to adults’ favoritism towards individuals with whom they have close ties. Nevertheless, multiple findings suggest that adults’ less favorable attitudes toward strangers, as opposed to familiar individuals, could also be alleviated through mentalization. Adults’ lesser willingness to help out-group members is associated with reduced abilities to mentalize about outgroups’ emotions [72]. When exposed to the suffering of out-groups in contrast to in-groups, adults exhibit reduced activity in brain areas linked to mentalizing, such as the medial prefrontal cortex and temporoparietal junction [e.g., 71, 73], as well as in the anterior insula (AI) and dorsal anterior cingulate cortex (dACC), regions often associated with affective empathy [74]. Recent evidence suggests that directing attention towards others’ emotions, as opposed to their cognitive processes, can effectively cultivate altruistic ideals such as benevolence [75] These findings support the idea that attending to others’ emotional experiences may have a significant role in cultivating prosocial behavior toward unfamiliar individuals, even among adults. Further research is required to validate this possibility.

The current findings also add to the previous literature suggesting a link between perspective-taking, empathy, and prosocial actions [51, 76]. Mentalizing their partner’s emotional state might have led the children to become more empathic, and thereby exhibit less favoritism toward familiar individuals. Emotional mentalization and empathy require an understanding of another person’s mental or emotional state; thus, they are positively related [77]. Although the current study did not measure children’s empathy levels, most children must have taken their partners’ emotional states into account. For example, in the emotional condition in Experiment 1, 31 of the 38 children answered the question “How sad did they feel?” with “very sad.” Further, in the negative emotional condition in Experiment 2, 30 of the 33 children provided the same response to the same question. These findings suggest that most children understood the protagonist’s emotions and might have experienced empathy. Similarly, recent meta-analytic research has revealed the activated areas shared by empathic processing and affective mentalizing, indicating a positive relationship between mentalizing and empathy [78]. These findings suggest that most children understood the protagonist’s emotions and might have experienced empathy.

Another crucial novel finding is that the impact of emotional mentalization on children’s sharing behavior varies depending on valence. In the current research, only exposure to another person’s negative emotional state mitigated bias toward friends. This result is consistent with previous findings that empathy for negative emotions is consistently more strongly correlated with prosociality than empathy for positive emotions [60, 68]. One possible explanation for this pattern is that children are more motivated to behave generously toward strangers who are in an unfavorable emotional state. Young children in particular are more likely to engage in prosocial behavior to alleviate another person’s negative emotional state [64]. Such tendencies may be related to negativity bias, which is the tendency to pay more attention to or be more influenced by negative emotion information than positive emotion information [e.g., 79, 80]. Researchers have found such negative bias in psychological reasoning early in infants [81, 82]. Furthermore, positive emotions are less attention-grabbing and lead to different social processing than negative emotions. Children who focus on another person’s positive emotions may experience an improvement in their emotions, reflecting self-interest rather than prosocial motivation [68]. Thus, empathizing with positive emotions may be associated with a mechanism that involves more selfish motivations.

Notably, that directing participants to think about their partner’s negative emotions increased the rate of equal distribution to strangers but not friends. At first, we were somewhat puzzled by this result, given previous evidence that promoting social interactions among same-gender children from the same kindergarten can increase equal sharing toward familiar others [83]. However, a close examination of the data suggests a potential ceiling effect for children’s equal distribution of resources to friends. In the control condition, a very high proportion of children (75% in Experiment 1 and 74% in Experiment 2) had already chosen to share a sticker with their friends, which might have been the upper limit on their generosity toward familiar others. One study also reported a similar level of bias toward familiar others in children’s sharing behaviors. Chinese children ages five to six years old exhibited significant in-group bias, with 75% of participants sharing equally with in-group members [62]. Therefore, it is not necessarily the case that our results are specific to strangers. Instead, the results indicate that focusing on another person’s negative emotions reduces bias toward friends by strengthening sharing intentions toward strangers. However, it is not clear whether sharing intentions toward friends could also be influenced by our mentalization manipulations.

Participants’ perceptions of the intensity of friends’ and strangers’ emotions did not differ significantly, regardless of emotional valence, suggesting that participants’ resource distribution choices were not a result of perceiving others’ emotions. Likewise, participants’ perceptions of their own emotions did not differ significantly across conditions. Thus, it is likely that focusing on others’ emotions did not promote prosociality through changes in the participants’ emotions or their perceptions of others’ emotions. Ultimately, the mechanisms of how attention to emotion reduces in-group bias are still unknown and could be examined in future research.

There may be questions regarding why the stories in Experiment 1 and 2’s emotional conditions employed an open-ended question rather than a Likert scale to obtain a fine-grained measure of the children’s emotion perceptions. We did not use a Likert scale for the priming question because the children’s responses to priming questions were not our main dependent variable of interest. More importantly, we wanted to prompt the children to consider the protagonist’s emotions more naturally. However, future research might utilize a Likert scale to effectively assess children’s perception of others’ emotional intensity while being primed. In addition, researchers might wish to examine the effect of children’s perceived emotions on mentalization about others’ psychological states.

The participants in the current research were Korean, which is considered a collectivist culture. Thus, the cultural context could have contributed to the current research findings, because children in collectivistic societies might exhibit stronger bias toward familiar others. Although no research has directly compared bias toward familiar others across different cultural groups, some prior evidence suggests that cross-cultural differences may exist in the development of such favoritism. For example, no evidence was found that five- to six-year-old European children showed in-group favoritism in resource distribution choices [13]. In contrast, five- to six-year-old Chinese children displayed significant in-group bias in resource distribution tasks—75% of participants shared equally with an in-group member, whereas only 44% did so with an out-group member [62]. It is worth noting that such numerical patterns are similar to those in the control condition in the current research—75% of participants shared equally with friends but only 33% did so with strangers in Experiment 1, and 74% shared equally with friends but only 39% did so with strangers in Experiment 2. Further cross-cultural research could investigate how cultural context influences the effect of the partner’s familiarity and emotional states on children’s sharing actions.

This research establishes that emphasizing another person’s negative emotional state may assist children in overcoming favoritism toward others with whom they have close ties in resource-distribution contexts. This study builds on previous research [25] by delving into the mechanisms underpinning mentalization inductions directed at unfamiliar others. Ultimately, in the current research, mentalizing about negative emotions did not impact the children’s perception of others’ emotions, but it did affect their willingness to alleviate others’ negative emotions through sharing actions, possibly mediated by enhanced empathy. Such findings align with the fundamental concepts of the widely accepted empathy-altruism hypothesis [84, 85]. In light of the meta-analytic finding that inducing emotions is strongly associated with minimizing prejudice between groups [48], our findings provide additional evidence that emotional mentalization–especially mentalizing about negative emotions–is effective in developing prosocial attitudes toward those with whom one does not have intimate ties. Our results have important implications for developing interventions to minimize interpersonal conflict and encourage prosocial behavior, hence enhancing peer relationships. Our research suggests that emotional understanding and prosocial motivation may be tightly connected, with children’s ability to comprehend the subjective internal states of others developing concurrently with prosocial motivation.

In addition, the present study’s results provide empirical evidence for the developmental process through which children gain knowledge about resource allocation. In the future, longitudinal studies in particular may help us better understand the durability of mentalization’s impact on children’s distributive behaviors toward familiar or unfamiliar others. Simultaneously, our study elucidates the factors affecting children’s prosocial distribution or sharing behavior, thereby improving moral performance. Finally, we hope that this study will provide useful insights for creating related child education programs.

## Supporting information

S1 Appendix Experimental instructions. (PDF)

S1 Data (PDF)
