# Supplementary material for: Focusing attention on others’ negative emotions reduces the effect of social relationships on children’s distributive behaviors
Source: PLoS One. 2024 Feb 7;19(2):e0295642. doi: 10.1371/journal.pone.0295642 (PMC10849392; doi:10.1371/journal.pone.0295642)

Supporting Information

Focusing Attention on Others' Negative Emotions Reduces the Effect of Social Relationships on Children's Distributive Behaviors

Minjung Cha[a] and Hyun-joo Song[a]

[a] Department of Psychology, Yonsei University, 50 Yonsei-ro, Seodaemun-gu, Seoul 03722,

Republic of Korea

Experimental Instructions

**The following instructions were translated into Korean and given to the experimenter.*

Example of the apparatus used in all experiments.

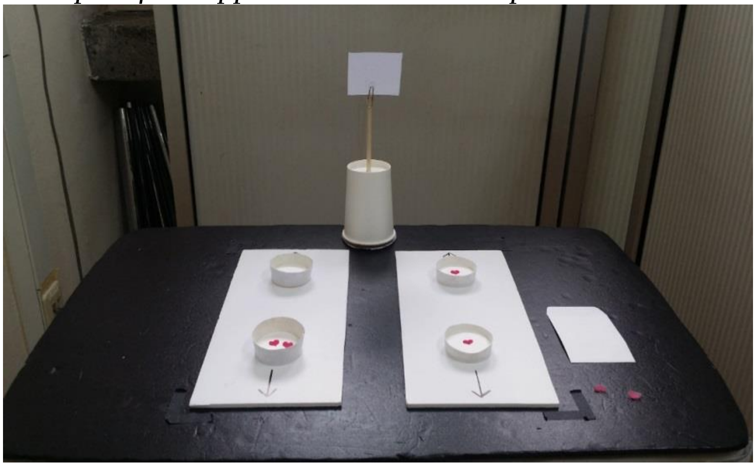

Experiment 1

Set	1st trial	2nd trial
A	Friend - 1:1 (L) 2:0 (R)	Stranger - 2:0 (L) 1:1 (R)
B	Stranger - 2:0 (L) 1:1 (R)	Friend - 1:1 (L) 2:0 (R)
C	Friend - 2:0 (L) 1:1 (R)	Stranger - 1:1 (L) 2:0 (R)
D	Stranger - 1:1 (L) 2:0 (R)	Friend - 2:0 (L) 1:1 (R)

<Settings>

1. Video recording and camcorder settings: Video recording is done with a Sony2 camera. The tripod to which the Sony2 camera is attached is placed on the edge of the TV desk so that the Experimenter (E) and the child can be seen on the screen.

2. Seating positions of the child and the researcher: The desk is turned on the left wall so that the E and the child can sit face to face.

3. Before the child enters the lab, in the waiting room, the E asks the child' parent, "Could I have the name of your child's best friend?" If the parent gives a name, the E confirms it with the child by asking, "Is _____ your best friend?" The E also asks the child, "Do you have any friends named Na-rae (if the child is a female) / Seok Yoon (if the child is a male)?"
The E then writes down the best friend's name on a nametag and "Narae (if female) / Yoon Seok (if male)" on another piece of paper. (If there is a Narae or Yunseok among the child's friend's names, the name is replaced by "Mihee (if female) / Dongsik (if male)." The E then makes up female/male names at their discretion until they find a name that is not among the child's friend's names.)

4. Before the start of the experiment, the E takes out the nametag and sets it in the marked place on the desk.

5. The sticker for the 1st trial is an orange heart sticker, and that for the 2nd trial is a dark pink star sticker.

6. After confirming that the setting is complete, the E writes the subject #, date of birth, and date of experiment on the whiteboard that has the experimental conditions written on it and records the experiment with the Sony2 camera.

7. After the E takes the time to get acquainted with the child in the waiting room, they go into the Preferential Looking Room with the child and place the child in the chair.

<1st TRIAL>

1. Introduction
"You are going to play a 'sticker game' with me and XX (the name of the best friend or stranger child). The nametag here says XX."

2. Introduction to the game
"Shall we play a sticker game together? In this game, I will give you two stickers.
(The E places two stickers on their hand and shows the child) "Look, here are two stickers."
(The stickers are not given to the child yet—the E keeps them.)

(The E presents the two sticker boards.)
"You can have two stickers like this (pointing to the right) or like this (pointing to the left). You can choose whatever you want out of these two, okay? The sticker or stickers you choose will be put in a paper bag later so you can take it or them home."

3. Mentioning the nametag
For Sets A and C: "XX (best friend's name) is your friend!

For Sets B and D: “XX (stranger’s name) is someone you don’t know!

4. Story Phase

Baseline condition

″[Best friend’s name or the stranger’s name] went to a store a few days ago because they wanted to buy some stickers. However, there weren’t any at that store, so they looked for another place to buy stickers and made a face like this (experimenter shows a picture of a neutral face to participant). Let’s think about where they searched for stickers (experimenter pauses for four seconds). Where do you think they went?”

Emotional condition

″[Best friend’s name or the stranger’s name] went to a store to buy some stickers a few days ago. However, there were no stickers left They felt very sad, so they made a sad face like this (the experimenter shows a picture of a sad face to the participant). Let’s think about how sad they felt for a second (experimenter pauses for four seconds). How sad did they feel?”

Cognitive condition

″[Best friend’s name or the stranger’s name] went to a store to buy some stickers a few days ago. However, there were no stickers left, so they thought about how to get the stickers. They made a thinking face like this (experimenter shows a picture of a neutral face to the participant). Let’s think about how much they thought about it for a second (experimenter pauses for four seconds). How hard do you think they thought?”

5. Distribution Phase

“So, how do you want to choose? (The E touches the right panel first.) Do you want to have it like this (right) or like this (left)?”

(When the child makes a choice, the E replies.) “OK. I will put the sticker(/s) in the paper bag and give it(/them) to you later.”

<2nd TRIAL>

For Sets A and C: Repeat the 1st trial for stranger

For Sets B and D: Repeat the 2nd trial for friend

Experiment 2

Set	1st trial	2nd trial
A	Friend - 1:1 (L) 2:0 (R)	Stranger - 2:0 (L) 1:1 (R)
B	Stranger - 2:0 (L) 1:1 (R)	Friend - 1:1 (L) 2:0 (R)
C	Friend - 2:0 (L) 1:1 (R)	Stranger - 1:1 (L) 2:0 (R)
D	Stranger - 1:1 (L) 2:0 (R)	Friend - 2:0 (L) 1:1 (R)

<Settings>

1. Video recording and camcorder settings: Video recording is done with a Sony2 camera. The tripod to which the Sony2 camera is attached is placed on the edge of the TV desk so that the Experimenter (E) and the child can be seen on the screen.

2. Seating positions of the child and the researcher: The desk is turned on the left wall so that the E and the child can sit face to face.

3. Before the child enters the lab, in the waiting room, the E asks the child' parent, "Could I have the name of your child's best friend?" If the parent gives a name, the E confirms it with the child by asking, "Is _____ your best friend?" The E also asks the child, "Do you have any friends named Na-rae (if the child is a female) / Seok Yoon (if the child is a male)?"
The E then writes down the best friend's name on a nametag and "Narae (if female) / Yoon Seok (if male)" on another piece of paper. (However, if there is a Narae or Yunseok among the child's friend's names, the name is replaced by "Mihee (if female) / Dongsik (if male)." The E then makes up female/male names at their discretion until they find a name that is not among the child's friend's names.)

4. Before the start of the experiment, the E takes out the nametag and sets it in the marked place on the desk.

5. The sticker for the 1st trial is an orange heart sticker, and that for the 2nd trial is a dark pink star sticker.

6. After confirming that the setting is complete, the E writes the subject #, date of birth, and date of experiment on the whiteboard that has the experimental conditions written on it and records the experiment with the Sony2 camera.

7. After the E takes the time to get acquainted with the child in the waiting room, they go into the Preferential Looking Room with the child and place the child in the chair.

<1st TRIAL>

1. Introduction
"You are going to play a 'sticker game' with me and XX (the name of best friend or stranger child). The nametag here says XX."
(The E presents the two sticker boards.)
"You can have these two stickers like this (pointing to the right) or like this (pointing to the left). You can choose whatever you want out of these two, okay? The sticker or stickers you choose will be put in a paper bag later so you can take it or them home."

3. Mentioning the nametag
For Sets A and C: "XX (best friend's name) is your friend!
For Sets B and D: "XX (stranger's name) is someone you don't know!

4. Story Phase
New Baseline condition
No story

Positive Emotional condition
"[Best friend's name or the stranger's name] went to the playground the other day and had so much fun with their friends. They felt so good and made a happy face like this (experimenter

shows a picture of a happy face to the participant). Let's think about how happy they were (experimenter pauses for four seconds). How happy do you think they were at this time?"

Negative Emotional condition

"[Best friend's name or the stranger's name] went to the playground the other day and did not have much fun with their friends. They felt so sad and made a sad face like this (experimenter shows a picture of a sad face to the participant). Let's think about how sad they were (experimenter pauses for four seconds). How sad do you think they were at this time?"

5. Distribution Phase
"So, how do you want to choose? (The E touches the right panel first.) Do you want to have it like this (right) or like this (left)?"
(When the child makes a choice, the E replies.) "OK. I will put the sticker(/s) in the paper bag and give it(/them) to you later."

6. Measuring Perceived Emotional Intensity
Positive Emotional condition

"[Best friend's name or the stranger's name] drew this picture a few days ago and was complimented by their teacher for their excellent drawing. How do you think they felt at that time?"

Participants respond to the final question using a four-point scale, which ranged from 1 (*"neither happy nor sad"*) to 4 (*"very happy"*).

Negative Emotional condition

"[Best friend's name or the stranger's name] drew this picture a few days ago, and someone tore the drawing when they were not looking. How do you think they felt at that time?"

Participants respond to the final question using a four-point scale, which ranged from 1 (*"neither happy nor sad"*) to 4 (*"very sad"*).

Examples of pictures used for Measuring Perceived Emotional Intensity

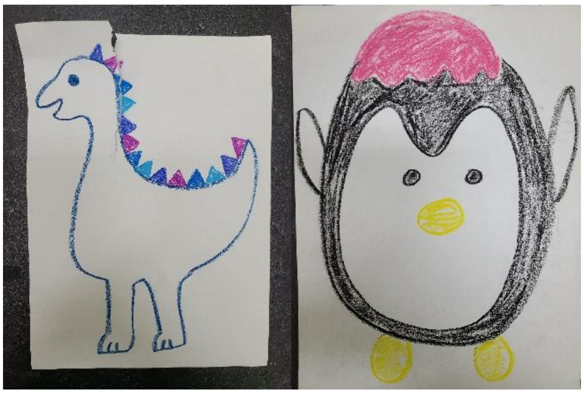

<2nd TRIAL>
For Sets A and C: Repeat the 1st trial for stranger
For Sets B and D: Repeat the 2nd trial for friend

Measuring Participant's Emotions
(The experimenter shows participants pictures of a happy face, a neutral face, and a sad face and asks them to choose a face consistent with their emotions.)

″Here are three faces showing different feelings: (The experimenter points to a picture of a happy face) this is a happy face, (The experimenter points to a picture of a neutral face) this face means that you are neither happy nor sad, (The experimenter points to a picture of a sad face) and this is a sad face. How do you feel right now? Can you point to the picture that describes your feelings?"

Example of the face scale used for Measuring Participant's Emotions

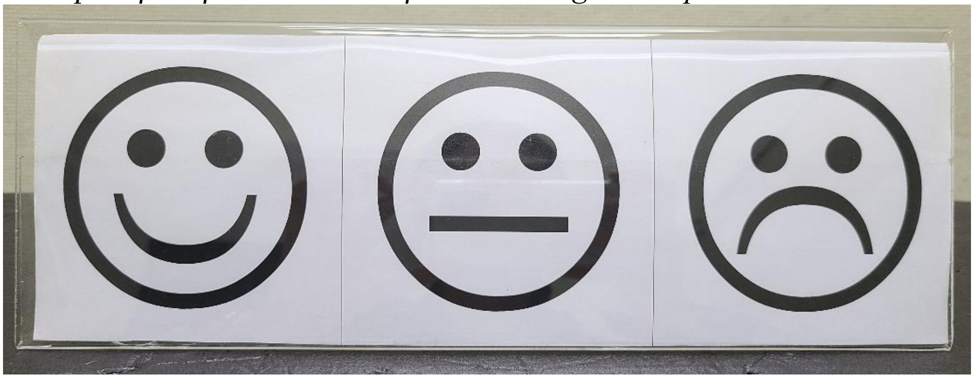

Supplement: S1 Appendix — (PDF) [file pone.0295642.s001.pdf]
