# Supplementary material for: Focusing attention on others’ negative emotions reduces the effect of social relationships on children’s distributive behaviors
Source: PLoS One. 2024 Feb 7;19(2):e0295642. doi: 10.1371/journal.pone.0295642 (PMC10849392; doi:10.1371/journal.pone.0295642)
Supplement: S1 Data — (PDF) [file pone.0295642.s002.pdf]

sub #	gender	age	experiment 1/2	condition	friend	non-friend
1	M	77.8	1	baseline	1	0
2	M	80.5	1	baseline	1	1
3	M	74.0	1	baseline	1	0
4	M	82.0	1	baseline	1	0
5	M	75.2	1	baseline	1	0
6	M	82.3	1	baseline	0	0
7	M	80.9	1	baseline	1	1
8	M	64.6	1	baseline	1	0
9	M	78.5	1	baseline	1	1
10	M	63.4	1	baseline	1	0
11	M	75.2	1	baseline	0	0
12	M	69.4	1	baseline	1	0
13	M	75.1	1	baseline	0	0
14	M	77.3	1	baseline	1	1
15	M	79.1	1	baseline	1	0
16	M	76.2	1	baseline	1	0
17	M	73.6	1	baseline	0	0
18	F	75.7	1	baseline	1	1
19	F	80.0	1	baseline	1	0
20	F	74.2	1	baseline	1	1
21	F	72.7	1	baseline	1	0
22	F	69.3	1	baseline	0	0
23	F	71.6	1	baseline	1	0
24	F	69.4	1	baseline	1	1
25	F	72.0	1	baseline	1	1
26	F	81.7	1	baseline	1	0
27	F	72.0	1	baseline	1	0
28	F	75.7	1	baseline	1	0
29	F	70.6	1	baseline	1	0
30	F	65.2	1	baseline	0	0
31	F	60.3	1	baseline	0	0
32	F	60.3	1	baseline	0	1
33	F	79.9	1	baseline	1	1
34	F	67.4	1	baseline	1	1
35	F	74.1	1	baseline	0	1

36	F	78.0	1	baseline	1	0
37	M	77.8	1	emotional	0	0
38	M	68.1	1	emotional	1	0
39	M	71.6	1	emotional	0	0
40	M	70.5	1	emotional	1	1
41	M	76.6	1	emotional	1	1
42	M	67.8	1	emotional	0	0
43	M	70.8	1	emotional	0	0
44	M	66.9	1	emotional	0	0
45	M	68.0	1	emotional	0	1
46	M	73.0	1	emotional	1	0
47	M	68.9	1	emotional	1	0
48	M	70.2	1	emotional	1	0
49	M	74.2	1	emotional	0	0
50	M	63.5	1	emotional	0	1
51	M	62.8	1	emotional	1	0
52	F	71.3	1	emotional	0	1
53	F	78.5	1	emotional	0	1
54	F	67.9	1	emotional	1	1
55	F	73.0	1	emotional	0	0
56	F	62.8	1	emotional	0	0
57	F	74.9	1	emotional	0	1
58	F	77.1	1	emotional	1	1
59	F	63.9	1	emotional	0	0
60	F	62.2	1	emotional	0	0
61	F	77.2	1	emotional	1	1
62	F	70.1	1	emotional	1	1
63	F	77.4	1	emotional	0	1
64	F	75.5	1	emotional	1	0

65	F	60.6	1	emotional	1	1
66	M	61.0	1	emotional	1	1
67	M	80.7	1	emotional	1	1
68	M	75.4	1	emotional	1	1
69	M	76.6	1	emotional	1	1
70	M	75.3	1	emotional	1	1
71	F	73.7	1	emotional	1	1
72	F	68.5	1	emotional	1	1
73	F	60.1	1	emotional	1	1
74	F	74.7	1	emotional	1	1
75	M	80.7	1	cognitive	1	1
76	M	61.4	1	cognitive	1	0
77	M	62.9	1	cognitive	1	1
78	M	66.9	1	cognitive	0	1
79	M	67.7	1	cognitive	0	1
80	M	80.0	1	cognitive	1	0
81	M	74.8	1	cognitive	1	0
82	M	71.9	1	cognitive	1	1
83	M	72.6	1	cognitive	0	0
84	M	68.1	1	cognitive	0	1
85	M	63.2	1	cognitive	0	0
86	M	63.2	1	cognitive	0	0
87	M	73.0	1	cognitive	1	1
88	M	74.1	1	cognitive	1	0
89	M	78.0	1	cognitive	1	0
90	F	74.1	1	cognitive	1	1
91	F	67.6	1	cognitive	1	0
92	F	73.2	1	cognitive	1	1
93	F	82.3	1	cognitive	1	1

94	F	66.0	1	cognitive	1	0
95	F	65.7	1	cognitive	1	0
96	F	66.8	1	cognitive	1	1
97	F	66.8	1	cognitive	1	0
98	F	73.2	1	cognitive	1	0
99	F	72.2	1	cognitive	1	1
100	F	76.8	1	cognitive	1	1
101	F	76.4	1	cognitive	1	1
102	F	70.4	1	cognitive	1	1
103	M	69.2	1	cognitive	0	0
104	M	76.7	1	cognitive	1	0
105	F	71.5	1	cognitive	1	1
106	F	73.3	1	cognitive	1	1
107	M	81.0	2	baseline	1	0
108	M	82.8	2	baseline	1	0
109	M	83.8	2	baseline	1	0
110	M	73.8	2	baseline	1	1
111	M	61.8	2	baseline	1	0
112	M	69.9	2	baseline	0	0
113	M	69.3	2	baseline	0	0
114	M	63.2	2	baseline	1	0
115	M	67.8	2	baseline	1	1
116	M	70.2	2	baseline	1	1
117	M	63.1	2	baseline	1	0
118	M	68.9	2	baseline	1	0
119	M	60.9	2	baseline	0	0
120	M	62.8	2	baseline	1	0
121	M	76.3	2	baseline	1	1
122	F	72.4	2	baseline	1	1

123	F	80.3	2	baseline	1	0
124	F	62.6	2	baseline	0	0
125	F	62.0	2	baseline	1	0
126	F	64.4	2	baseline	1	1
127	F	71.2	2	baseline	1	0
128	F	64.8	2	baseline	0	0
129	F	71.3	2	baseline	1	1
130	F	63.8	2	baseline	0	1
131	F	74.6	2	baseline	0	1
132	F	71.0	2	baseline	1	1
133	F	64.6	2	baseline	1	1
134	F	63.9	2	baseline	0	0
135	F	63.8	2	baseline	1	1
136	F	73.5	2	baseline	1	0
137	F	61.6	2	baseline	1	0
138	M	75.8	2	positive emotional	0	0
139	M	80.1	2	positive emotional	1	0
140	M	77.5	2	positive emotional	1	0
141	M	73.9	2	positive emotional	1	0
142	M	72.1	2	positive emotional	0	1
143	M	73.2	2	positive emotional	1	1
144	M	70.6	2	positive emotional	0	0
145	M	73.9	2	positive emotional	0	0
146	M	72.0	2	positive emotional	0	0
147	M	82.1	2	positive emotional	1	1
148	M	69.7	2	positive emotional	1	0
149	M	72.9	2	positive emotional	1	1
150	M	62.1	2	positive emotional	0	0
151	M	75.4	2	positive emotional	1	0

152	M	75.5	2	positive emotional	1	1
153	F	79.4	2	positive emotional	1	1
154	F	69.3	2	positive emotional	0	0
155	F	76.8	2	positive emotional	1	0
156	F	79.7	2	positive emotional	1	0
157	F	74.9	2	positive emotional	1	1
158	F	76.0	2	positive emotional	0	0
159	F	71.1	2	positive emotional	0	0
160	F	70.9	2	positive emotional	1	0
161	F	77.6	2	positive emotional	0	1
162	F	75.2	2	positive emotional	1	1
163	F	65.1	2	positive emotional	1	1
164	F	60.6	2	positive emotional	0	0
165	F	73.1	2	positive emotional	1	0
166	F	64.4	2	positive emotional	0	0
167	F	76.3	2	positive emotional	1	0
168	F	73.9	2	positive emotional	1	1
169	M	64.0	2	negative emotional	0	0
170	M	61.1	2	negative emotional	0	0
171	M	83.5	2	negative emotional	1	1
172	M	61.3	2	negative emotional	0	0
173	M	73.5	2	negative emotional	1	0
174	M	76.4	2	negative emotional	0	1
175	M	79.1	2	negative emotional	1	1
176	M	80.0	2	negative emotional	1	1
177	M	77.7	2	negative emotional	0	1
178	M	66.9	2	negative emotional	0	1
179	M	72.1	2	negative emotional	1	1
180	M	72.1	2	negative emotional	0	0

181	M	74.7	2	negative emotional	1	1
182	M	62.0	2	negative emotional	0	0
183	M	83.8	2	negative emotional	1	1
184	M	66.7	2	negative emotional	1	1
185	F	65.5	2	negative emotional	0	0
186	F	65.7	2	negative emotional	0	0
187	F	75.4	2	negative emotional	1	1
188	F	61.8	2	negative emotional	1	1
189	F	63.8	2	negative emotional	1	0
190	F	68.4	2	negative emotional	1	1
191	F	77.7	2	negative emotional	0	1
192	F	70.7	2	negative emotional	1	0
193	F	78.6	2	negative emotional	1	0
194	F	78.4	2	negative emotional	1	1
195	F	61.8	2	negative emotional	1	1
196	F	61.9	2	negative emotional	1	1
197	F	74.6	2	negative emotional	1	1
198	F	79.8	2	negative emotional	1	1
199	F	77.7	2	negative emotional	1	1
200	F	71.3	2	negative emotional	1	1
201	F	81.0	2	negative emotional	1	1
